# Behavioral Characterization of *dmrt3a* Mutant Zebrafish Reveals Crucial Aspects of Vertebrate Locomotion through Phenotypes Related to Acceleration

**DOI:** 10.1523/ENEURO.0047-20.2020

**Published:** 2020-05-18

**Authors:** Ana del Pozo, Remy Manuel, Ana Belen Iglesias Gonzalez, Harmen Kornelis Koning, Judith Habicher, Hanqing Zhang, Amin Allalou, Klas Kullander, Henrik Boije

**Affiliations:** 1 Department Neuroscience, Uppsala University, S-75124 Uppsala, Sweden; 2 Department of Information Technology, Division of Visual Information and Interaction, Uppsala University, S-75105 Uppsala, Sweden; 3 BioImage Informatics Facility, SciLifeLab, S-75105 Uppsala, Sweden

**Keywords:** central pattern generator, *Danio rerio*, gait, locomotion, spinal cord, wt1

## Abstract

Vertebrate locomotion is orchestrated by spinal interneurons making up a central pattern generator. Proper coordination of activity, both within and between segments, is required to generate the desired locomotor output. This coordination is altered during acceleration to ensure the correct recruitment of muscles for the chosen speed. The transcription factor *Dmrt3* has been proposed to shape the patterned output at different gaits in horses and mice. Here, we characterized *dmrt3a* mutant zebrafish, which showed a strong, transient, locomotor phenotype in developing larvae. During beat-and-glide swimming, mutant larvae showed fewer and shorter movements with decreased velocity and acceleration. Developmental compensation likely occurs as the analyzed behaviors did not differ from wild-type at older larval stages. However, analysis of maximum swim speed in juveniles suggests that some defects persist within the mature locomotor network of *dmrt3a* mutants. Our results reveal the pivotal role Dmrt3 neurons play in shaping the patterned output during acceleration in vertebrates.

## Abstract (precis)

Vertebrate locomotion is orchestrated by spinal interneurons making up a central pattern generator. Proper coordination of activity, both within and between segments, is required to generate the desired locomotor output. This coordination is altered during acceleration to ensure the correct recruitment of muscles for the chosen speed. The transcription factor *Dmrt3* has been proposed to shape the patterned output at different gaits in horses and mice.

## Significance Statement

This study shows that *dmrt3a*-expressing spinal neurons are crucial for coordinating locomotion in fish, a function that must have arisen early during the evolution of vertebrates. Analyses of two *dmrt3a* mutant zebrafish lines, one similar to the “gait-keeper” mutation in horses and one similar to the null mutant in mice, allow us to evaluate differences in locomotor phenotypes within a single species. Characterization throughout development gives insights into the fundamental role these interneurons play to establish coordinated locomotion. Our results suggest that zebrafish is an excellent model to reveal how speed changes are orchestrated in vertebrates by examining the activity of *dmrt3a*-expressing interneurons *in vivo* at a cellular level in relation to acceleration.

## Introduction

Animals display diverse limb and axial locomotor gaits when moving at different speeds. A central pattern generator (CPG), composed of spinal interneurons, orchestrates locomotion by dictating frequency output, left/right alternation, and intersegmental coordination (for review, see Boije and Kullander, 2018). A speed-dependent modularity of the CPG has been evidenced in several vertebrates, such as humans, mice, and fish (McLean et al., 2008; Talpalar et al., 2013; Ampatzis et al., 2014; Yokoyama et al., 2016). This has been particularly well described in zebrafish, where motor neurons, V2a and V0 interneurons, as well as muscles, have been categorized into speed-dependent modules. Adult zebrafish exhibit three modules (slow, intermediate, and fast), which are sequentially recruited as swim speed increases (Ampatzis et al., 2014; Björnfors and El Manira, 2016), while larvae only display the slow and fast modules (McLean et al., 2008). These modules in zebrafish translate into different movement regimes (i.e., gears); at slow swim speeds, zebrafish larvae use both fins and axial body undulations, while only the axial body is involved during fast swim speeds (Green and Hale, 2012). Speed is also modulated within a single gear, where larvae vary the bout duration, interbout period, and tail amplitude during slow swim, while the tail beat frequency is altered during fast swim (Severi et al., 2014). Hence, zebrafish locomotion requires changes in the coordination within and between spinal cord segments while accelerating, by recruiting different speed modules. It is not yet known how these changes are coordinated during increased speeds.

Doublesex and mab-3-related transcription factor 3 (*Dmrt3*) is expressed in the developing spinal cord and takes part in fate specification of dI6 interneurons (Andersson et al., 2012). These interneurons have been shown to coordinate locomotion in horses and mice (Andersson et al., 2012; Perry et al., 2019). A truncating mutation in *Dmrt3* was found to underlie the two additional gaits, pace and tölt, performed by Icelandic horses adding to the three natural gaits (walk, trot, and gallop; Andersson et al., 2012). This mutation has been found in multiple horse breeds with additional gaits, which suggest that it is permissive of alternative limb coordination (Promerová et al., 2014). *Dmrt3*-null mice displayed impaired limb coordination in neonates, with disturbed left/right alternation as well as uncoordinated front/hindlimb movements (Andersson et al., 2012). Moreover, Dmrt3 neurons are active at variable rhythms during fictive locomotion, which suggests that the locomotion may be affected by the activity from Dmrt3 neurons at various speeds (Perry et al., 2019). A recent study in larval zebrafish showed that *dmrt3a*-expressing neurons are rhythmically active during locomotion and that they provide mid-cycle inhibition onto contralateral motor neurons (Satou et al., 2020).

A fate switch likely occurs in the spinal cord of *Dmrt3*-null mice as there is an increase in the number of cells expressing the transcription factor Wilms’ tumor 1 (*Wt1*), a marker for a subpopulation of dI6 progenitors, at the expense of *Dmrt3*-expressing cells (Andersson et al., 2012). Similarly, when *Wt1* is inactivated in mice, a fate switch from dI6 to V0-like neurons takes place and the number of *Dmrt3*-expressing neurons is reduced, which further suggests an interplay between of these two transcription factors in fate determination (Schnerwitzki et al., 2018).

Analysis of *Dmrt3*-null mice suggests altered regulation of *Dmrt1* but not *Dmrt2* in the absence of *Dmrt3* (Andersson et al., 2012). In vertebrates, *Dmrt1*, *Dmrt3*, and *Dmrt2* are clustered in a conserved order in the genome, a region that did not undergo the extra round of genome duplication in teleost fish (Brunner et al., 2001; Johnsen and Andersen, 2012). As in mammals, *dmrt3a* is expressed by neurons in the developing spinal cord of zebrafish (Li et al., 2008); however, their impact on locomotor behavior is unknown.

To investigate whether *dmrt3a*-expressing interneurons coordinate locomotion in fish, we performed behavioral analyses on *dmrt3a* mutant zebrafish. We found that a truncating mutation, predicted to resemble a null mutant, showed strong defects in larvae locomotion. In-depth behavioral analysis revealed defects in parameters related to acceleration, swim speed, and tail kinematics. Overall, our findings highlight the role of *dmrt3a*-expressing neurons in the coordination of locomotion in fish, a functional trait that seems to be conserved among vertebrates.

## Materials and Methods

### Experimental design

*Animals.* All adult zebrafish used in this study were of the AB strain and housed at the Genome Engineering Zebrafish National Facility (SciLifeLab, Uppsala, Sweden) under standard conditions of 14/10 h light/dark cycles at 28°C. Embryos and larvae for the experiments were obtained from group breeding and were kept under constant darkness at 28°C until 6 d postfertilization (dpf). Larvae tested at 10 or 22 dpf and 6-week-old juveniles were housed from 6 dpf onward under the same housing conditions as adults. Sex was not determined for experimental animals. Appropriate ethical approvals were obtained from a local ethical board in Uppsala (C 164/14).

**Figure 1.**
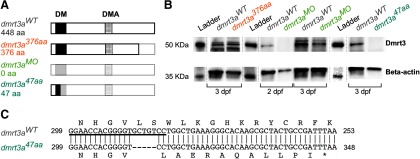
Description of the zebrafish models used. ***A***, Schematic structure of the predicted Dmrt3a protein, showing the DNA binding domain (DM) and the Dmrt3 family domain (DMA). Lighter shade represents missing amino acids compared with the wild-type form of Dmrt3a. ***B***, Western blot for Dmrt3a (47 kDa) and β-actin (42 kDa) protein at 3 dpf in *dmrt3a^WT^*, *dmrt3a^47aa^*, and *dmrt3a^376aa^* as well as 2 and 3 dpf in *dmrt3a^MO^*. ***C***, Alignment of *dmrt3a* cDNA partial sequences between *dmrt3a^WT^* (top) and the CRISPR/Cas9 generated mutant *dmrt3a^47aa^*(bottom). The fragment shows the proximity to the 5 bp deletion (—) where the asterisk (*) represents the premature stop codon generated and the underlined sequence indicates the sgRNA target. This figure is extended in Extended Data Figure 1-1.

Three different models (Fig. 1*A*) were used in this study, and experimental animals were always compared with their respective wild-type (WT) siblings (all three referred to as *dmrt3a^WT^*). Only homozygote mutants were used in the experiments.

10.1523/ENEURO.0047-20.2020.f1-1 Figure 1-1 Alignment of *dmrt3a* cDNA from *dmrt3a^47aa^*and *dmrt3a^WT^.* Colors highlight the relevant sites for the generation of *dmrt3a^47aa^*, *dmrt3a^MO^*, and *dmrt3a^376aa^*. Download Figure 1-1, TIF file.

A zebrafish line, which carries the allele *sa15557*, was acquired from the Zebrafish Mutation Project (Kettleborough et al., 2013; referred to as *dmrt3a^376aa^*). In this model, a single nucleotide polymorphism in *dmrt3a* (T > A at 1333 of 1920 bp, including UTR regions) introduces a premature stop codon in the second exon of the *dmrt3a* gene. The putative protein would have 376 aa (out of 448 aa), still encoding the DNA binding domain and the Dmrt family domain, but missing the sequence after the second domain (Fig. 1*A*).

To generate Dmrt3a knock-down morphants (referred to as *dmrt3a^MO^*), a translation blocking morpholino (MO) antisense oligo for *dmrt3a* RNA (5′-GGGCGATCCGTAGCCATTCATTTCT-3′) was obtained from Gene Tools (Philomath) alongside standard control MO (5′-CCTCTTACCTCAGTTACAATTTATA-3′). Wild-type zebrafish were divided into two groups and then yolk injected at the one-cell stage with 2 ng of *dmrt3a* MO or standard control MO.

A *dmrt3a* mutant zebrafish line, carrying the allele *UU232* (referred to as *dmrt3a^47aa^*), was generated by the Genome Engineering Zebrafish National Facility (SciLifeLab, Uppsala University, Sweden), using the CRISPR/Cas9 technique described in the study by Varshney et al. (2015). The single guide RNA (sgRNA) target used to induce site-specific gene alterations was GGAACCACGGGGTGCTGTCC (Fig. 1*C*, Extended Data Fig. 1-1). A 5 bp deletion was found by fragment length analyses on the PCR product (forward M13F-tailed primer, 5′-TGTAAAACGACGGCCAGTTACGGATCGCCCTACCTCTA-3′; reverse PIG-tailed primer, 5′-GTGTCTTAAACTCTCGTTCGCCTGCT-3′; and M13F-FAM) and was decoded by sequencing. The deletion occurred at 313–317 bp (including 5′UTR) and introduced a premature stop codon in the first exon at 353–355 bp. This model has a putative protein of 47 aa (out of 448 aa), where only the first 36 aa are identical to the native protein (Fig. 1*A*,*C*, Extended Data Fig. 1-1). Thus, only a small part of the DNA binding domain (12 of 46 aa) would be properly coded.

*Western blot.* Zebrafish embryos were collected at 3 dpf for *dmrt3a^47aa^* and *dmrt3a^376aa^*, and at 2 and 3 dpf for *dmrt3a^MO^*, alongside their respective *dmrt3a^WT^*. Approximately 20 embryos were pooled per sample, and the yolk sac was removed in deyolking buffer (Ringer’s solution without calcium and with EDTA) as described in the study by Westerfield (2007). Samples were homogenized in RIPA buffer with protease inhibitor (Thermo Fisher Scientific). The Western blot was performed as described by Blixt et al. (2018) with minor modifications. Proteins were separated on a 10% Mini-Protean TGX Stain-Free Precast gel (Bio-Rad) for 40 min at 200 V and transferred to a Supported Nitrocellulose Membrane (Bio-Rad) for 1 h at 100 V. The following primary antibodies were used: Dmrt3, 1:2000, rabbit (Invitrogen) and β-actin, 1:2000; mouse (Sigma-Aldrich). The following secondary antibodies were used: rabbit IgG-HRP conjugated, 1:3000 (Bio-Rad); and mouse IgG-HRP conjugated, 1:3000 (Bio-Rad). Ladder lanes were separated from the rest of the membrane and incubated with Precision Protein StrepTactin-HRP Conjugate (Bio-Rad) for chemiluminescent detection. This was done to avoid unspecific binding by this kit on the samples. Membranes (including ladder) were incubated for 5 min in Western ECL Substrate (Bio-Rad) and chemiluminescence was detected by ChemiDoc MP Imaging System (Bio-Rad). The relative expression of Dmrt3a protein detected by the Western blot analysis was quantified using Fiji (version 1.52i; Schindelin et al., 2012). To do so, pixel intensity in the 8 bit Western blot image was measured within equal-sized ROIs for each protein (Dmrt3a or β-actin) and each lane background. After subtracting the lane background from each band intensity, relative expression of the Dmrt3a band was calculated in relation to the β-actin band for each sample. Finally, a comparison was made with the corresponding controls.

*Free swimming and escape response in larvae.* The free locomotor behavior for all three models (*dmrt3a^47aa^*, *dmrt3a^376aa^*, and *dmrt3a^MO^*) was tested in separated trials, and compared with their corresponding *dmrt3a^WT^*. Mutant/morphant and control larvae were individually distributed in 48-well plates in a checker board pattern. These larvae were housed in their respective wells from 3 to 6 dpf, after which the larvae were moved into the nursery system. At 10 and 22 dpf, larvae were collected and tested in 24-well and 6-well plates, respectively. Larvae were imaged using Noldus DanioVision (Noldus Information Technology) zebrafish hardware system at 25 frames per second (fps), where larvae were acclimatized for 20 min in white light at 28°C. The following two consecutive trials were executed: (1) free-swimming trial where larvae were exposed for another 50 min to the same conditions; and (2) an escape trial where a sequence of five taps was executed with 3 min intervals. This spacing has been reported to be sufficient to avoid habituation to the taps (Issa et al., 2011).

The tracking was performed by Noldus EthoVision XT video tracking software (version 13, Noldus Information Technology) using dynamic subtraction as the detection method. Thus, Ethovision collected samples (i.e., data of larvae coordinates) at every frame, information that was later computed into the desired parameters. Several parameters were analyzed over the whole 50 min free-swimming trial: total displacement, number of movements, mean movement duration, mean time per acceleration, mean velocity while moving, maximum acceleration, and maximum velocity. For the escape response, the following variables were analyzed over the 280 ms after each tap: maximum acceleration, maximum velocity, cumulative time accelerating, total displacement, and cumulative velocity over the escape response. Then an average for the five taps for each larvae was calculated. For visualizing the parameters dynamic, values were also extracted in bins of 10 min for the free-swimming trial and bins of 40 ms for the escape trial. No statistical analyses were performed on individual bins.

*Tail kinematics in larvae.* To analyze tail movements at high temporal resolution, *dmrt3a^47aa^*and *dmrt3a^WT^* larvae were individually semiconstrained in 1.2% low melt agarose (Sigma-Aldrich), where the head was embedded but the tail was free. Bouts were recorded under a stereoscope (model MZ10F, Leica) at 1000 fps by a high-speed camera (TS4, Fastec Imaging). Tail movements were tracked using the Python scripts from Semmelhack et al. (2014), with minor modifications. Briefly, 20 points homogeneously distributed along the tail were tracked throughout the recording. Following that, a self-developed Python script was used to score tail kinematics from the tracked data. To eliminate the noise from tail drifting, the coordinates were smoothed by a mobile average over 15 frames and three tail points.

For the parameters described below, bouts and half-beats were defined according to Marques et al. (2018); half-beats were classified as swimming episodes or escape responses based on tail deflection (<35°or >35°, respectively); slow and fast swimming half-beats were cataloged by half-beat frequencies <60 Hz or >60 Hz, respectively; “tail deflection” is the angle between the imaginary line of the tail base-to-tip and the vertical; “tail curvature” is the sum of all absolute angles between consecutive segments along the tail; “tail trajectory” is the displacement of the tail tip in the horizontal axis; and “tail velocity” is the tail trajectory divided by the half-beat period. All parameters were calculated separately for fast and slow half-beats. Among the analyzed parameters were the following: the number of half-beats during the bout, bout duration, cumulative and mean tail trajectory over the bout, mean tail velocity over the bout, maximum deflection over the bout, maximum curvature over the bout, and variance of period between half-beats over the bout.

*Spontaneous coiling in embryos.* We first established the coiling dynamics during development by assessing spontaneous coiling in unhatched WT zebrafish embryos between 17 and 28 h postfertilization (hpf). Groups of 15 embryos were recorded for 5 min every hour at 50 fps using a high-speed camera (model TS4, Fastec Imaging) mounted on a stereoscope (model MZ10F, Leica). Using the same methodology, *dmrt3a^47aa^* and *dmrt3a^WT^* were filmed in mixed groups between 19 and 22 hpf, followed by genotyping. Videos were processed in Fiji (version 1.52i; Schindelin et al., 2012). Individual embryos were assigned ROIs, and movements were detected by subtracting the pixel intensity of the previous frame using the Stack Difference plugin. The difference in pixel intensity between frames was exported as a text document for data processing in Excel 2010 (Microsoft; see Fig. 4*A*,*B*). A custom template detected and characterized the peaks of movement, reporting coiling frequency, coil duration, and coil intensity among other parameters.

*Maximum swim speed in juveniles.* The maximum swim speed of 6-week-old *dmrt3a^47aa^*zebrafish was determined by subjecting the fish to a water flow of increasing speeds. Zebrafish were acclimatized to the experimental facility at room temperature for 2 weeks before the trial. *dmrt3a^47aa^*and *dmrt3a^WT^* were individually placed in a 10 L swim tunnel (Loligo Systems) with a 30 × 10 × 10 cm swim arena. The protocol to determine the maximum swim speed (Umax) of zebrafish was based on the study by Gilbert et al. (2014). Briefly, zebrafish in the swim tunnel were firstly acclimatized at a low flow speed (4.5 cm/s) for 5 min at 28°C. Then, water velocity was increased by 4.5 cm/s in 1 min steps. Failure was defined as the moment fish were forced against the rear of the test section for >5 s, and the maximum speed was then set as the last step before failure. In total, three fish from the *dmrt3a^47aa^*group never initiated swimming (i.e., remained resting at the bottom) under experimental conditions and were excluded from the experiment. Fish length was measured from the tip of the mouth to the end of the peduncle and used to convert the maximum speed to body lengths per second. We observed no significant differences (*t* = 0.6332, df = 121, *p* = 0.53) in body length between *dmrt3a^WT^* (0.91 ± 0.09, *n* = 64) and *dmrt3a^47aa^* (0.92 ± 0.10, *n* = 59).

*Quantitative PCR.* The mRNA expression of *dmrt1a*, *dmrt2a*, *dmrt3a*, and *wt1a* was quantified in *dmrt3a^47aa^ and dmrt3a^WT^*from 1 to 5 dpf. The tails of dechorionated embryos and larvae were collected and homogenized in Invitrogen TRIzol (Thermo Fisher Scientific). RNA was extracted by isopropanol–ethanol precipitation according to Green and Sambrook (2017). The relative expression of analyzed genes was normalized to two housekeeping genes, *rpl13* and *elfa*, using the δ-δ Ct method (Vandesompele et al., 2002). Internal controls, introduced to each quantitative PCR (qPCR) plate, allowed for comparison between trials. The primers used are listed in Table 1.

**Table 1:** Primer sequences for qPCR or to create probes for *in situ* hybridization

Test	*Gene*	Reference	Primer	Primer sequences (5′–3’)
mRNA in situ hybridization	*dmrt1a*	AY157562.1	F R	GGCCACAAACGCTTCTGTAA TTGTAACTGGCAGCTGGAGA
	*dmrt2a*	NM_130952.1	F R	ATCCACCCAGTCCAACTCAG TTCCTCCAGCAGCTCCTTAC
	*dmrt3a*	NM_001005779.2	F R	CTCTGGCACCTTTGGAAACC TTGTGGGCAGGGAAGATCTT
	*wt1a*	NM_131046.1	F R	TGACCCAACTTGACTTTGCG CTGGAGGAGGAACGGGATAC
qPCR	*dmrt1a*	AY157562.1	F R	GCCAGTGTCAGAAATGCAGA TGAACCGGAAAGGTTAATCG
	*dmrt2a*	NM_130952.1	F R	CCCGCGATTTGTAATGTGGC GAGGGTGACTTTCGGTGGAG
	*dmrt3a*	NM_001005779.2	F R	TGGCAGTGACAGAGAACCAG GGGTCAGAGCAGGATTTTGA
	wt1a	NM_131046.1	F R	GGAAGTCAAGCTCTGCTGCT AACCTCCTGGATGGCTCTTT
	*elfa*	AY422992	F R	CTTCTCAGGCTGACTGTGC CCGCTAGCATTACCCTCC
	*rpl13*	NM_212784	F R	TCTGGAGGACTGTAAGAGGTATGC AGACGCACAATCTTGAGAGCAG

F, Forward; R, reverse.

*Whole-mount* in situ *hybridization.* The mRNA expression pattern of *dmrt1a*, *dmrt2a*, *dmrt3a*, and *wt1a* was visualized in *dmrt3a^47aa^ and dmrt3a^WT^*larvae. In order to inhibit pigmentation, 0.003% 1-Phenyl-2-thiourea was added to the embryo water at 24 hpf. Zebrafish were collected at 3 dpf and fixed overnight using 4% paraformaldehyde in PBS at 4°C, then washed once in 100% methanol for 15 min and stored in 100% methanol at −20°C till use. When unhatched, embryos were manually dechorionated before fixation. A cDNA library from pooled larvae (1–5 dpf) was generated by the QuantiTec Reverse Transcription Kit (Qiagen) and used as a template in a reverse transcription with specific primers containing a T7 overhang (Table 1) for each gene of interest. The specific cDNA templates were purified using a cDNA Purification Kit (Qiagen) and were used for probe synthesis by T7 RNA polymerase (Sigma-Aldrich) and labeled with digoxigenin-labeled UTP (Digoxigenin RNA Labeling Kits, Roche) according to the manufacturer instructions. mRNA probes were purified using the Invitrogen RNAeasy Purification Kit (Thermo Fisher Scientific). mRNA probes were stored in 50% formamide at −20°C till use. *In situ* hybridization was performed as previously described in the study by Thisse and Thisse (2008) with the addition of permeabilization of the larvae with Proteinase K at 5 μg/ml (BD Biosciences) for 20 min. Probes were used at a concentration of 50 ng/200 μl hybridization buffer. Hybridized probes were visualized using BM Purple (Roche) as a substrate for alkaline phosphatase precipitation. Embryos were cleared in 99% glycerol.

We used a custom-built Optical Projection Tomography (OPT) system for imaging of the *in situ* stained zebrafish embryos (Sharpe et al., 2002). The OPT system, reconstruction algorithms, and alignment workflow are based on the methods described in Allalou et al. (2017). Therotational images are acquired using a 3× telecentric objective with a pixel resolution of 1.15 μm/pixel. The tomographic 3D reconstruction was performed using a filtered back projection algorithm in MATLAB (Release R2015b; MathWorks) together with the ASTRA Toolbox (van Aarle et al., 2015). For the alignment of the data, we use the registration toolbox elastix (Klein et al., 2010; Shamonin et al., 2014). In the registration, all 3D volumes are downsampled two times to reduce the computational time. For each *in situ* probe, we generated two average volumes, one for *dmrt3a^WT^* and one for *dmrt3a^47aa^*. Each average is generated from five samples using an Iterative Shape Averaging (ISA) algorithm (Rohlfing et al., 2001). The initial registration in the ISA was performed with a rigid transform followed by an affine. The final average patternwas done using a b-spline deformable transform. To be able to compare the average expression patterns, the *dmrt3a^WT^* and *dmrt3a^47aa^* averages for each probe were aligned to each other.

### Statistical analyses

All values are given together with the SEM. Each experimental group by developmental stage was compared with their respective control group. Statistical analyses and the plotting of data were performed by SPSS statistical software version 25 (SPSS). Data were first tested for normality by Shapiro–Wilk test, and equality of variances was assessed with a Levene’s test. Unpaired Student’s *t* tests were applied in case of parametric data and a Mann–Whitney *U* test for two independent samples in case of nonparametric data. The level of significance was set at *p *<* *0.05 (two-tailed). All the statistical values, *p* values and the number of trials and animals used for each assay are included in Extended Data Figure 2-1 and Extended Data Figure 3-1.

**Figure 2.**
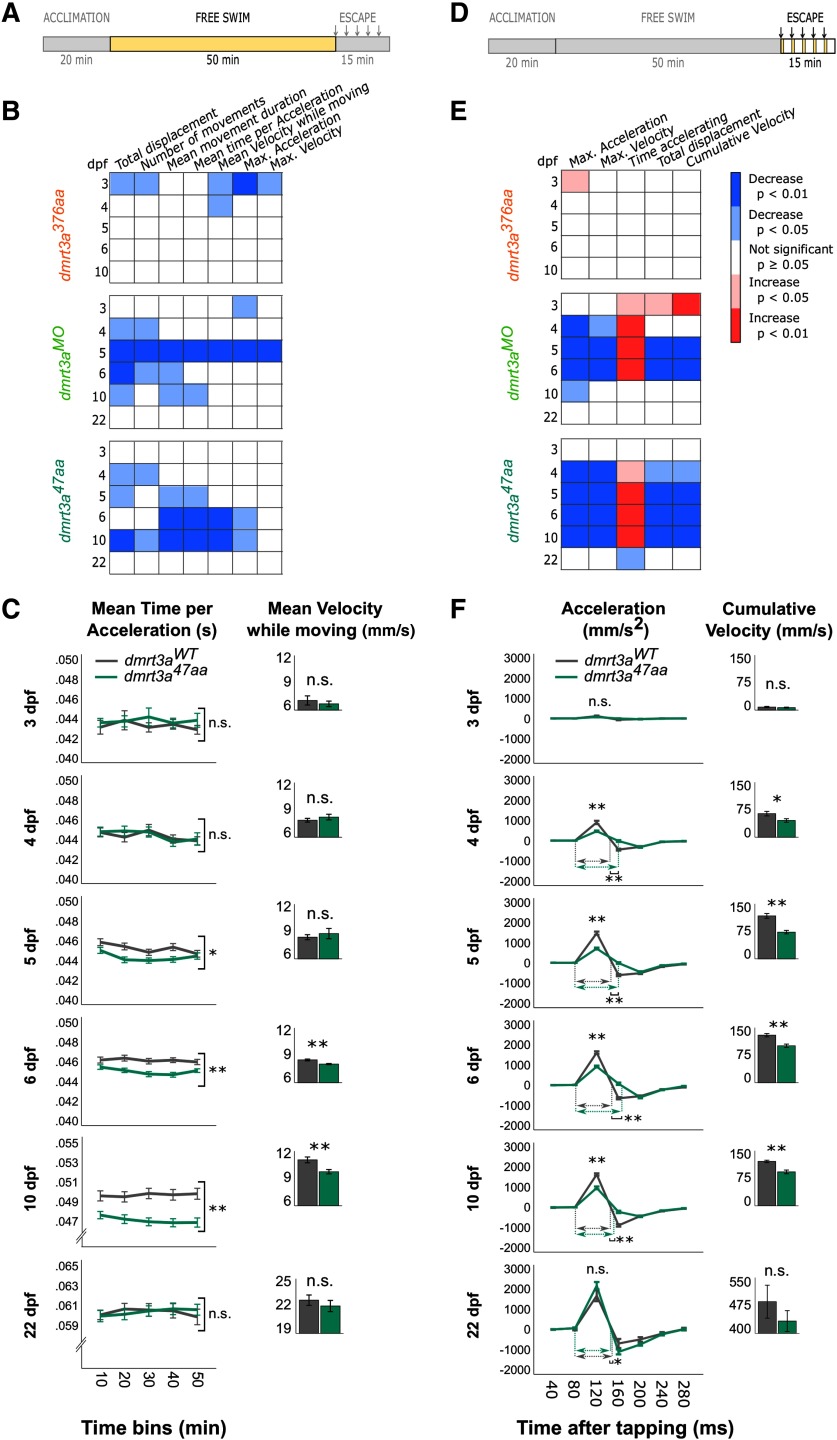
Comparative locomotor analyses of the three zebrafish models (*dmrt3a^376aa^*, *dmrt3a^MO^*, and *dmrt3a^47aa^*) in relation to their *dmrt3a^WT^*. ***A***, ***D***, Schematic of experimental protocol indicates whether data below belong to the free swimming (***A***) or to the escape response (***D***); arrows designate timing of taps. ***B***, ***E***, Heat maps to visualize statistical differences among *dmrt3a^376aa^*, *dmrt3a^MO^*, and *dmrt3a^47aa^* in relation to their *dmrt3a^WT^* during 1–6 and 10 dpf over the 50 min of free swimming (***B***) and over the 280 ms of induced escape responses (***E***). Increase (red) and decrease (blue) of parameters in mutant larvae and morphants compared with *dmrt3a^WT^*. ***C***, Mean time per acceleration of *dmrt3a^47aa^* and their *dmrt3a^WT^* are represented in 10 min bins during free swimming for a dynamic visualization over the trial. However, statistics are performed on the whole trial. Inset, Bar graphs show the mean velocity while moving over the trial. ***F***, Acceleration dynamic of *dmrt3a^47aa^* and their *dmrt3a^WT^* during escape response in 40 ms bins. Inset, bar graphs show cumulative velocity over the escape. Dashed horizontal arrows indicate the duration of the acceleration phase (in ms) for each group when they are significantly different. All data are plotted as the mean ± SEM. *p < 0.05. **p < 0.01. n.s. - not statistically significant. Statistical data from this figure are shown in Extended Data Figure 2-1.

10.1523/ENEURO.0047-20.2020.f2-1 Figure 2-1 Number of replicates, statistical values, and *p* values for all the variables, developmental stages, and zebrafish models assessed during free swimming and escape trials. Download Figure 2-1, DOCX file.

**Figure 3.**
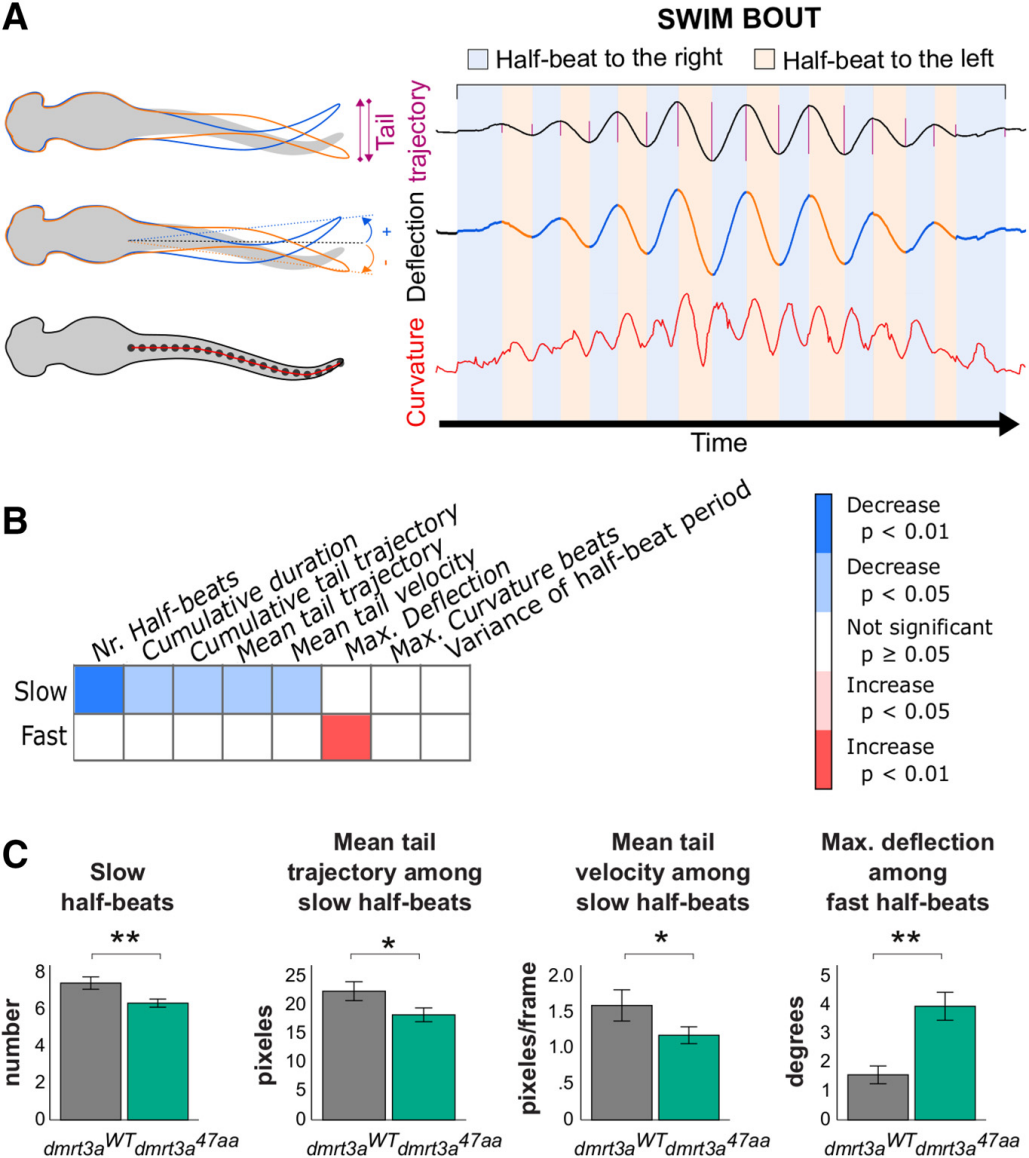
Tail kinematics in *dmrt3a^47aa^* animals. ***A***, Representative trace of a larva, illustrating the definition of parameters analyzed, such as tail trajectory, deflection, and curvature. ***B***, Heat map to visualize statistical differences between 6 dpf *dmrt3a^47aa^* and *dmrt3a^WT^*, during both slow and fast half-beats, separately. The increase (red) and decrease (blue) of parameters in mutant larvae are compared with *dmrt3a^WT^*. ***C***, Most relevant parameters where *dmrt3a^47aa^* differed from *dmrt3a^WT^* (i.e., the number of slow half-beats, mean tail trajectory among the slow half-beats, mean tail trajectory, and mean tail velocity among the slow half-beats, and the maximum deflection among the fast half-beats). All data are plotted as the mean ± SEM. *p < 0.05. **p < 0.01. Statistical data from this figure are shown in Extended Data Figure 3-1.

10.1523/ENEURO.0047-20.2020.f3-1 Figure 3-1 Number of replicates, statistical values, and *p* values for all the variables assessed during coiling, tail kinematics, and maximum swim speed. Download Figure 3-1, DOCX file.

## Results

### Larvae with a late truncating mutation show a minor locomotor phenotype

The “gait-keeper” mutation in *Dmrt3*, initially found in Icelandic horses, occurs toward the end of the gene, and the conserved DNA and protein binding domains are retained (Andersson et al., 2012). We analyzed zebrafish larvae with a similar mutation (i.e., the *dmrt3a^376aa^*), which carries a point mutation introducing a premature stop codon relatively close to the end of the coding region (Fig. 1*A*). Thus, *dmrt3a^376aa^* produces a putative Dmrt3 protein of 376 aa, ∼8 kDa smaller than the WT protein. Dmrt3 protein from *dmrt3a^376aa^* was detected by Western blot using an antibody that recognizes amino acids 341–375 (Fig. 1*B*). In the free swim analysis, mutant larvae showed a minor locomotor phenotype, but only at the earliest stages analyzed (3–4 dpf); a time period when zebrafish display rudimentary locomotor activity (Fig. 2*A*,*B*). At 3 dpf, larvae are only able to perform slow swim bursts (<40 Hz); however, at 4 dpf they start to swim in a beat-and-glide manner that becomes more frequent and sustained at 5 dpf (Berg et al., 2018). Mutant larvae moved less frequently and with reduced maximum acceleration and velocity at 3 dpf (Fig. 2*B*). Moreover, their mean velocity during movements was lower at both 3 and 4 dpf. During escapes, *dmrt3a^376aa^* larvae differed from their siblings only with respect to their maximum acceleration at 3 dpf, which showed a small increase (Fig. 2*D*,*E*). For all other parameters and ages, there were no significant differences between *dmrt3a^376aa^* larvae and their control siblings.

### Translation-blocking morpholinos targeting *dmrt3a* mRNA induced a clear locomotor phenotype

Considering the weak locomotor phenotype of *dmrt3a^376aa^* larvae, we decided to disrupt the production of Dmrt3a protein by injecting translation-blocking morpholinos (Fig. 1*A*,*B*). Western blot analysis verified a reduction in the abundance of Dmrt3a protein in relation to *dmrt3a^WT^* (91% at 2 dpf and 53% at 3 dpf; Fig. 1*B*). These morphants showed a distinct locomotor phenotype. During free swimming, morphants differed from their controls in a few parameters at 3–4 dpf, more parameters decreased at 5 dpf, after which the phenotype started to fade and eventually disappeared at 22 dpf (Fig. 2*A*,*B*). Affected locomotor parameters included the following: diminished movements, shorter acceleration bouts, and reduced maximum velocity and acceleration. The locomotor phenotype was more evident and uniform during the escape responses (Fig. 2*D*,*E*). Although, there were no differences in the number of larvae that responded to mechanical taps between morphants and their controls (data not shown), *dmrt3^MO^* larvae performed differently than their controls from 4–6 dpf. These differences included reduced maximum acceleration and velocity and a prolonged duration of acceleration (Fig. 2*D*,*E*).

### Characterization of an early truncating mutation in *dmrt3a* exposed transient motor defects

Since off-target effects of morpholinos may be a concern (Stainier et al., 2017), and the transient nature of our morphant phenotype may indicate reduced efficiency of the morpholino; a stable loss-of-function mutant (*dmrt3a^47aa^*) was generated to verify the phenotype observed in *dmrt3a^MO^*. The *dmrt3a^47aa^* zebrafish carry a premature stop codon in the beginning of the *dmrt3a* gene, resulting in a truncated protein of 47 aa (instead of 448 aa) where the last 11 aa are frame shifted (Fig. 1*A–C*). Western blot analysis at 3 dpf verified the absence of Dmrt3a protein (Fig. 1*B*).

The analysis of free swimming during early development revealed diminished activity in *dmrt3^47aa^* larvae (Fig. 2*A–C*). The *dmrt3a^47aa^* larvae showed locomotor deficits at 4 dpf, when beat-and-glide swimming appears. The phenotype strengthened, affecting more parameters in 6–10 dpf larvae, when swimming is more sustained. The effects mainly consisted in a reduction of the number and/or duration of movements, and a shortening of the time spent accelerating. Maximum acceleration and mean velocity while moving were also decreased. However, similar to *dmrt3a^MO^*, *dmrt3a^47aa^* larvae showed no locomotor phenotype in free swimming at 22 dpf. Analysis of the escape response revealed a phenotype in 4–10 dpf *dmrt3a^47aa^* larvae, with a lower maximum acceleration and velocity but an increased acceleration time (Fig. 2*D–F*). Although mutant larvae had a prolonged acceleration phase, the total/cumulative velocity and distance moved was reduced. Nevertheless, by 22 dpf, the only significant difference observed was a slightly decreased acceleration time.

As both *dmrt3a^MO^* and *dmrt3a^47aa^* larvae showed robust locomotor phenotypes at 6 dpf, this age was chosen to perform a more detailed analysis of tail movements. Semiconstrained larvae rarely reached the fast swim speeds of free-swimming animals. Hence, the majority of analyzed tail half-beats were classified as slow movements. Our analysis of 6 dpf *dmrt3a^47aa^* larvae revealed differences during slow half-beats (<60 Hz; Fig. 3*A–C*). We observed fewer half-beats per bout, reduced mean tail trajectory, and decreased mean tail velocity compared with *dmrt3a^WT^* (Fig. 3*B*,*C*). Although fast half-beats (>60 Hz) seldom occurred in the swim bouts we recorded, they did reveal that *dmrt3a^47aa^* larvae had a greater maximum deflection over the bout.

**Figure 4.**
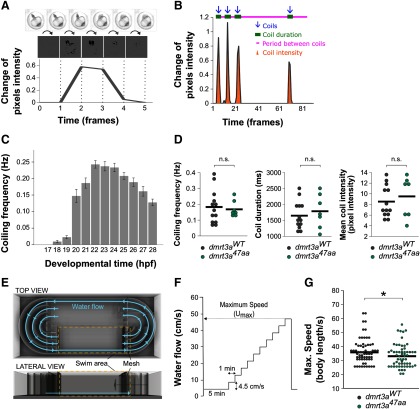
Locomotor activity of *dmrt3a^47aa^* embryos and juveniles. ***A***, Coil movement detection by quantifying pixel change from frame to frame. ***B***, A representative analysis and extraction of parameters. Detected coils (blue arrows), coiling duration (green bars), intercoil duration (pink line), and coil intensity (orange area) were quantified for each individual. ***C***, Coil frequency (Hz) in wild-type zebrafish from 17 to 28 hpf. ***D***, Coiling frequency (Hz), duration (ms), and coil intensity (pixel intensity) of *dmrt3a^47aa^* and *dmrt3a^WT^*. n.s., *p* ≥ 0.05. ***E***, Schematic of swim tunnel used for analysis in juveniles: top and lateral views. ***F***, Experimental protocol applied to determine maximum speed (U_max_). ***G***, Maximum swim speed in body lengths per second reached by the *dmrt3a^47aa^* and *dmrt3a^WT^*. Individual data are represented by dots, and the mean is indicated by a horizontal line. *p < 0.05. n.s. - not statistically significant. Statistical data from this figure are shown in Extended Data Figure 3-1.

The first locomotor activity of zebrafish embryos consists of spontaneous slow alternating coil movements of the tail. Coiling behavior in WT embryos first occurred at 18 hpf. These movements became prominent at 20 hpf and reached a maximum frequency at 22 hpf (Fig. 4*A–C*). Hence, *dmrt3a^47aa^* and *dmrt3a^WT^* embryos, were observed from 20 to 22 hpf. Embryos with the *dmrt3a^47aa^* mutation did not differ in any of the studied parameters, such as frequency of movements, mean coiling duration, or mean coil intensity (Fig. 4*D*).

We next turned to 6-week-old juveniles, which swim in consecutive episodes during which the amplitude of the body undulations decreases (Müller et al., 2000; Gabriel et al., 2008). After acclimation at 4.5 cm/s for 5 min, the water velocity was increased by 4.5 cm/s in 1 min steps until the fish failed to maintain swim (Fig. 4*E*,*F*). The maximum swim speed was significantly lower for the *dmrt3a^47aa^* mutants (median, 34.4 body lengths/s) compared with *dmrt3a^WT^* (median, 32.1 body lengths/s; Fig. 4*G*).

This suggests that although the strong locomotor phenotype observed in young developing larvae fades as the circuit matures, there are still coordination defects leading to a reduced performance in juvenile *dmrt3a^47aa^* zebrafish.

### Analysis of *dmrt1a*, *dmrt2a*, *dmrt3a*, and *wt1a* revealed differential expression in *dmrt3a^47aa^* larvae

Analysis of *Dmrt3*-null mice suggests altered regulation of *Dmrt1* but not *Dmrt2* in the absence of *Dmrt3* (Andersson et al., 2012). *Dmrt3*-null mutant mice also showed an increase in the abundance of *Wt1*-expressing neurons, a subpopulation formed from the same progenitor domain (dI6). We therefore performed whole-mount *in situ* hybridization and qPCR to analyze the expression of these genes in *dmrt3a^47aa^* and *dmrt3a^WT^*.

**Figure 5.**
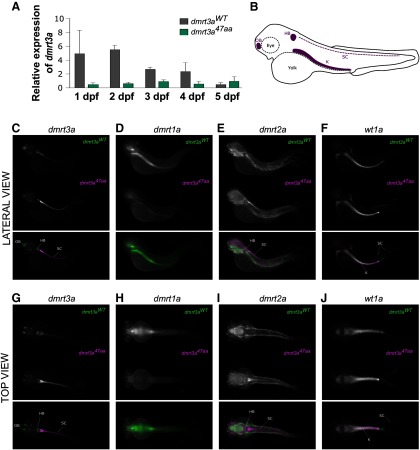
Expression of *dmrt3a* and related genes in *dmrt3a^47aa^* and *dmrt3a^WT^*. ***A***, Relative expression of *dmrt3a* mRNA from 1 to 5 dpf. ***B***, Schematic of a 3 dpf larva where relevant structures are labeled in purple [i.e., hindbrain (HB), spinal cord (SC), olfactory bulb (OB), and kidney (K)]. ***C–J***, mRNA pattern expression at 3 dpf. Top and lateral views: images of *dmrt3a^47aa^* and *dmrt3a^WT^* represent the average expression of 5 larvae. Merged color images below show the expression from *dmrt3a^WT^* in green and from *dmrt3a^47aa^* in magenta. Top view: ***C***, *dmrt3a* expression; ***D***, *dmrt1a* expression; ***E***, *dmrt2a* expression; ***F***, *wt1a* expression. Lateral view: ***G***, *dmrt3a* expression; ***H***, *dmrt1a* expression; ***I***, *dmrt2a* expression; ***J***, *wt1a* expression. Abbreviations highlight relevant labeling. This figure is extended in Extended Data Figure 5-1.

To quantify and map the developmental expression of *dmrt3a*, we performed qPCR on 1–5 dpf embryos. Expression of *dmrt3a* was highest at 1–2 dpf in *dmrt3a^WT^* and decreased in older embryos. In contrast, expression levels of *dmrt3a* were lower in *dmrt3a^47aa^* compared to *dmrt3a^WT^* and remained unchanged during development (Fig. 5*A*). Unfortunately, the expression of *dmrt1a*, *dmrt2a*, and *wt1a* could not be quantified accurately in the spinal cord due to contamination of nonspinal tissues. Expression of *dmrt2a* has been described in somites and muscles, while *wt1a* is strongly expressed in the developing kidney (Winkler et al., 2004; Bollig et al., 2009). Practical limitations prevented us from avoiding these structures while collecting the spinal tissue. Still, qPCR was performed on these genes and revealed a loss of *dmrt1a* expression in *dmrt3a^47aa^* larvae (Extended Data Fig. 5-1*A–C*).

10.1523/ENEURO.0047-20.2020.f5-1 Figure 5-1 Relative mRNA expression of *dmrt1a*, *dmrt2a*, and *wt1a* from 1 to 5 dpf *dmrt3a^47aa^*and *dmrt3a^WT^*. Data are plotted as the mean ± SD. *N* = 3, where 10–30 pooled larvae per *N*. No statistical analysis was performed. ***A***, *dmrt1a* relative expression. ***B***, *dmrt2a* relative expression. ***C***, *wt1a* relative expression. Download Figure 5-1, EPS file.

*In situ* hybridization revealed labeling for *dmrt3a* mRNA in the olfactory bulbs, and hindbrain, and along the spinal cord of both *dmrt3a^WT^* and *dmrt3a^47aa^* larvae (Fig. 5*B*,*C*,*G*). The expression of *dmrt1a* was detected in the brain and hindbrain of *dmrt3a^WT^* larvae, but not in the spinal cord (Fig. 5*D*). No staining was found for *dmrt1a* in *dmrt3a^47aa^* animals (Fig. 5*H*). Expression of *dmrt2a* was detected in the brain and muscle for *dmrt3a^WT^*, but not the spinal cord (Fig. 5*E*). Interestingly, *dmrt3a^47aa^* animals showed stained tissue in the hindbrain and the start of the spinal cord (Fig. 5*I*). We observed labeling for *wt1a* mRNA in the kidney and hindbrain, and at the end of the spinal cord in both *dmrt3a^47aa^* and *dmrt3a^WT^* larvae (Fig. 5*F*,*J*).

## Discussion

The spinal locomotor CPG modulates the coordination within and between segments to recruit the proper muscles when an animal changes its gait. A mutation in the transcription factor *Dmrt3* enables alternative gaits in horses and results in disturbed locomotor coordination in null mice (Andersson et al., 2012). To investigate the role of *dmrt3a* within the locomotor network of zebrafish, we performed behavioral analyses on zebrafish mutants. The loss of *dmrt3a* affected acceleration and tail coordination in larvae and reduced the maximum speed in juveniles.

### The gait-keeper mutation produces a mild locomotor phenotype in zebrafish

Analyses of *dmrt3a^376aa^*, hosting a mutation that resembles the gait-keeper mutation found in horses, only revealed a mild locomotor phenotype (Fig. 2*B*). The zebrafish mutation occurs slightly further down the coding region than in horses but both retain the Dmrt family binding domain. The strong penetrance of a locomotor phenotype in horses, even in heterozygous animals, suggests that the truncated protein acts as a dominant negative (Andersson et al., 2012). The lack of a persistent phenotype in *dmrt3a^376aa^* zebrafish could be due to an inability of the truncated Dmrt3a to function as a dominant negative. However, it should be noted that horses require extensive training for alternative gaits to arise. Foals carrying the *Dmrt3* mutation have not been reported to have locomotor defects, and some breeds require years of training to bring forth unnatural gaits. Hence, lack of training may prevent our fish from developing altered locomotor coordination.

### Loss of Dmrt3a protein during zebrafish development resulted in disturbed motor behavior

Translation-blocking morpholinos rendered an altered locomotor behavior in both spontaneously free-swimming zebrafish larvae and during induced escape bouts (Fig. 2*B*,*E*). Parameters related to velocity and acceleration were reduced in 5–6 dpf *dmrt3a^MO^* larvae, while older animals displayed a milder phenotype or no phenotype. Analysis of a loss-of-function mutant (*dmrt3a^47aa^*), lacking DNA-binding and Dmrt family domain, reproduced the morphant locomotor phenotype.

No effect was observed on the earliest embryonic movements, the spontaneous coils generated by the rudimentary CPG (Fig. 4*D*). These types of early movements, which enable the maturation of the network, have not been analyzed in *Dmrt3* mutant mice, hence a comparison cannot be made. Although *dmrt3a*-expressing neurons exist at the time of coiling, they are either not functional or not critical to perform the movement.

Analysis of free-swimming zebrafish revealed that both *dmrt3a^47aa^* and *dmrt3a^MO^* initiated fewer and shorter swim movements, and that the fish traveled a shorter distance (Fig. 2*B*,*C*). In addition, these animals spent less time accelerating and had a lower maximum acceleration. Although the consequence of these deficits affected the average velocity, there was little effect on the maximum velocity. This suggests that these animals are less likely to initiate swim movements and have problems accelerating to faster swim. The initiation requires the onset of alternating contractions and tail amplitude is used to modify speed within the slow swim, whereas the acceleration to fast swim transfers the focus to the tail beat frequency (Severi et al., 2014). The phenotype observed may relate to coordination defects within slow/fast swim movements, to the transition between the two, or to both. Null mice pups showed reduced alternation pointing to disturbed coordination within a segment; while adults displayed defects related to flexor/extensor coordination, revealing disturbance in intersegmental communication (Andersson et al., 2012). These defects in mice could relate to the phenotype observed in zebrafish as both the initiation and coordination of a structured locomotor output would be affected.

When escape bouts were elicited, the locomotor deficiencies described above became more apparent. The *dmrt3a^47aa^* larvae could not accelerate as fast or reach the same maximum velocity as *dmrt3a^WT^* during these short reflex-induced bouts. Interestingly, the time spent accelerating increased, maybe in an attempt to compensate for the reduced acceleration (Fig. 2*E*,*F*). However, it may also be linked to a reduced ability to transition from fast to slow swim movements.

Both *dmrt3a^47aa^* and *dmrt3a^MO^* showed a lack of locomotor phenotype at 22 dpf, suggesting compensatory mechanisms during development. The apparent transient nature of the phenotype is well in line with mouse data where locomotor coordination improves in older animals (Andersson et al., 2012). Although our detailed behavioral analysis at 22 dpf showed no locomotor phenotype, the analysis of maximum performance in juveniles suggests that locomotor defects remain in advanced stages. This too is in line with the mouse data, where null mutants failed to run at high speeds (Andersson et al., 2012).

### Detailed tail analysis provides explanations to the observed defects

Tail kinematics analysis of semiconstrained *dmrt3a^47aa^* larvae revealed fewer half-beats per bout during slow swim, which correlates with the shorter acceleration observed during the free swimming (Fig. 3*B*,*C*). We also observed a reduced tail trajectory and lower tail speed during slow swim, which may explain the decreased acceleration and velocity observed in free-swimming animals. Analysis of fast movements showed an increase in the maximum deflection of the tail in *dmrt3a^47aa^* compared with *dmrt3a^WT^*. This may be a consequence of difficulties in the transition between left–right alternations, where the inertia could produce a greater deflection angle. Similarly, *Dmrt3*-null mice elongated their stride when forced to run at high speeds (Andersson et al., 2012). By increasing the phase duration of the swing and stance, the mice may attempt to compensate for the aberrant coordination, which prevents them from reaching a higher locomotor frequency. Fictive locomotion experiments on larval zebrafish, which had their *dmrt3a*-expressing neurons genetically ablated, revealed an increase in left/right coactivation of the same segment (Satou et al., 2020). This coordination defect may explain the altered tail movements observed in the current study.

### Candidate genes for compensatory mechanisms

The *Dmrt* genes are located in tandem in vertebrategenomes and their close proximity, with overlapping regulatory regions and conserved protein domains, could result in compensatory mechanisms in mutants (El-Mogharbel et al., 2007). The three genes are known to be coexpressed during gonad development, a feature conserved among vertebrates (Brunner et al., 2001).

*In situ* hybridization revealed *dmrt3a* expression in the olfactory bulb, the hindbrain, and the spinal cord in both *dmrt3a^WT^* and *dmrt3a^47aa^* animals, indicating that the cells still developed in the absence of Dmrt3a (Fig. 5*C*,*G*). However, qPCR suggested that *dmrt3a* mRNA levels are reduced as a consequence of the mutation (Fig. 5*A*). Expression of *dmrt1a* and *dmrt2a* has not been reported in the spinal cord of zebrafish, and the expression of *dmrt2* is absent in the developing spinal cord of medaka fish (Winkler et al., 2004). In accordance, our *in situ* hybridization did not reveal *dmrt1a* expression in the spinal cord; however, expression was seen in regions of the head (Fig. 5*D*,*H*). Interestingly, this expression was lost in *dmrt3a^47aa^* larvae, making it unlikely that *dmrt1a* compensates for the absence of Dmrt3a (Fig. 5*H*, Extended Data Fig. 5-1*A*). Expression of *dmrt2a* has been described in somites and muscles, but not in spinal cord (Winkler et al., 2004). In line with these findings, the expression of *dmrt2a* was not detected in the spinal cord of *dmrt3a^WT^*; however, staining was observed for *dmrt2a* in the spinal cord of *dmrt3a^47aa^* animals, indicating an upregulation (Fig. 5*E*,*I*). Coinjection of *dmrt3a* and *dmrt2a* translation blocking morpholinos rendered a locomotor phenotype identical to that of *dmrt3a^MO^*, suggesting that there is no functional compensation (data not shown). These observations for *dmrt1a* and *dmrt2a* are in contrast to what has been reported in *Dmrt3*-null mice, where there is an upregulation of *Dmrt1* but not *Dmrt2* in the absence of *Dmrt3*. This discrepancy may be explained by differences in genomic rearrangement between mouse and fish models (Andersson et al., 2012). As previously reported (Bollig et al., 2009), w*t1a* was strongly expressed in the developing kidney (Fig. 5*F–J*). *In situ* data also revealed a larger area of *wt1a* expression in the spinal cord for *dmrt3a^47aa^* larvae, compared with *dmrt3a^WT^*. In light of the rostral-to-caudal maturation of the spinal cord, it is impossible to deduce whether the caudal *wt1a* expression was due to a developmental shift (delayed/prolonged) or increased fate assignment. Because of the strong expression of *wt1a* in nonspinal tissues, we were unable to accurately quantify the expression by qPCR for the spinal cord. The mouse data, which showed an increase in the number of Wt1-positive cells, also failed to discriminate whether this was a result of developmental delay or increased fate assignment (Andersson et al., 2012). Further studies are needed to unravel the formation of *dmrt3a*-expressing and *wt1a*-expressing neurons in the spinal cord.

### Conclusion

We demonstrated the importance of *dmrt3a*-expressing neurons in the coordination of locomotion in fish. Behavioral analyses in *dmrt3a^376aa^*, *dmrt3a^47aa^*, and *dmrt3a^MO^* allowed us to compare the effects of different alterations at different developmental stages in a single animal model, shedding light on the divergences between the Icelandic horses and the *Dmrt3*-null mice locomotor phenotypes. Differences in phenotypes between the models may be due to the developmental stages at which behavior was analyzed, the genetic differences that underlie their behavioral phenotypes, or the training capacity of horses. The possibility of dominant-negative actions of the truncated horse protein, with a high penetrance in heterozygotes, and the lack of phenotype in heterozygous mice and fish (data not shown) suggest that a combination of genetics and training underlie the observed locomotor differences.

This study, confirming the involvement of *dmrt3a*-expressing neurons in zebrafish locomotion, is critical in the pursuit to unravel the function of this important regulatory component of the CPG. *Dmrt3* mutant mice showed uncoordinated output regarding left/right alternation and flexor/extensor muscles, suggesting that locomotor defects arise as a result of miscommunication both within and between spinal segments (Andersson et al., 2012). We draw a similar conclusion and hypothesize that Dmrt3 neurons could help to facilitate the change between speed modules, coordinate locomotion within a module, or both. A recent study by Satou et al. (2020) indicates that *dmrt3a*-expressing neurons have an increased firing probability during slow speeds, which may result in inhibition of the fast module. It will be crucial to study the firing properties of individual *dmrt3a* neurons and connect their activity to the locomotor output, with a focus on the speed-dependent submodules and the transitions between them.

## Synthesis

Reviewing Editor: Jeffery Twiss, University of South Carolina Decisions are customarily a result of the Reviewing Editor and the peer reviewers coming together and discussing their recommendations until a consensus is reached. When revisions are invited, a fact-based synthesis statement explaining their decision and outlining what is needed to prepare a revision will be listed below. The following reviewer(s) agreed to reveal their identity: Tuan Bui. Note: If this manuscript was transferred from JNeurosci and a decision was made to accept the manuscript without peer review, a brief statement to this effect will instead be what is listed below.

The analysis of zebrafish Dmrt3a morphants and mutants suggest specific changes in the speed and acceleration of swimming movements in early development that subside later in life, highlighting the need to better understand the role of these neurons in locomotor circuits. The reviewers noted the advantage of the zebrafish over mammalian models for the wide range of techniques available and the ease and rapidity of generating mutants, thus allowing us presumably to decipher how dmrt3 mutation impairs the cells and its networks important for locomotion. Overall, the reviewers’ felt that the revised manuscript addresses most of their comments and the reviewing editor agrees. However, there are still 2 lingering issues that the reviewers and feel need to be addressed before further consideration of the manuscript. I append the reviewer’s comments below as they are quite clear, but please also note my points from the online discussion below and take these into consideration for revising your manuscript:

1. The statistics used in Fig 2C need a better description for rationale and method (Reviewer 1, point 2).

2. The authors do a good job of characterizing the swimming behavior of developing Dmrt3 morphant and mutant zebrafish, but there is a lack of information and quantification regarding anatomical, synaptic, and molecular changes in these mutants that would strengthen the current study (Reviewer 2, point 1). With regards to this point, the reviewer suggests IHC as an alternative approach. However in our online discussion, the possibility that specific antibodies may not exist and, if they do exist, may not work in zebrafish (also they may be quite difficult to obtain at the present time). Reviewer 2 pointed out that your ISH is working “but the resolution is insufficient to get any valuable information from the whole mount preparations. Therefore, could the authors quantify ISH signals from spinal cord sections instead? Also, could they combine ISH with axonal tracing to quantify commissural and projecting dmrt3 interneurons from spinal cord sections of mutant larvae or juveniles?” Our online discussion also raised that intraspinal tract tracing is challenging in zebrafish but that you may have other techniques to provide some idea of projection (e.g., plasmids, transgenics).

Reviewer 1:

1. The Intro and Discussion should be amended to take into consideration a recent paper from the lab of Shinichi Higashijima (Satou et al. 2020 Cell Reports) where they investigated the role of Dmrt3a+ dI6 neurons in swimming at different speeds. Their work also showed that these Dmrt3a+ dI6 neurons, along with V0d neurons, form the morphologically defined CoBL neurons as identified by Liao and Fetcho in 2008. The results of their work should be compared to the results of this study in the intro and discussion

2. For Fig 2C, it is still not clear how the tests were done on the whole swimming trial rather than on a bin-per-bin basis. That is, parameters such as mean time per acceleration are tested during the whole trial rather than in individual bins is not clear in terms of how this is accomplished. This is something that was picked up by the other reviewer, but what was added in the figure legend by the authors, does not explain the mechanics of this statistical test. Was there some sort of integration done on the data in each bin to come up with a single value per trial?

Reviewer 2:

1. The authors’ attempts to quantify the expression of dmrt3a, 1a, 2a, and wt1 by in situ hybridization or qPCR are valuable, but unfortunately were unsuccessful. As mentioned in my initial review, this is likely due to an ectopic expression of these genes outside of the spinal circuit but also a lack of specificity inherent to these techniques. As an alternative, IHC staining of spinal tissues should be considered for qualitatively and quantitatively analyzing whether transient behavioral changes parallel some transient or permanent anatomical changes that could explain functional compensations through development.

2. There are conjugation and grammar errors in the paper. The draft will need to be proofread by a native English speaker.

Minor comments

Line 63: Remove the last sentence. If these last studies suggest an interplay between Dmrt3 and wt1 in fate determination, there is no information regarding changes in the properties of Dmrt3 neurons, only in the number of cells.

Line 356: the statement that CPG “alters” coordination makes it sound as if it is a disease or impairment. Replace “alters” by “modulates.”

Line 370: what do the authors mean by “training is required to allow for alternative gaits to arise” regarding dmrt3376aa zebrafish?

Line 420: add “phase duration” to “swing and stance.”

Line 462: add locomotion in “developing fish.”
