# Supplementary figures and images for: Behavioral Characterization of dmrt3a Mutant Zebrafish Reveals Crucial Aspects of Vertebrate Locomotion through Phenotypes Related to Acceleration
Source: eNeuro. 2020 May 18;7(3):ENEURO.0047-20.2020. doi: 10.1523/ENEURO.0047-20.2020 (PMC7235372; doi:10.1523/ENEURO.0047-20.2020)

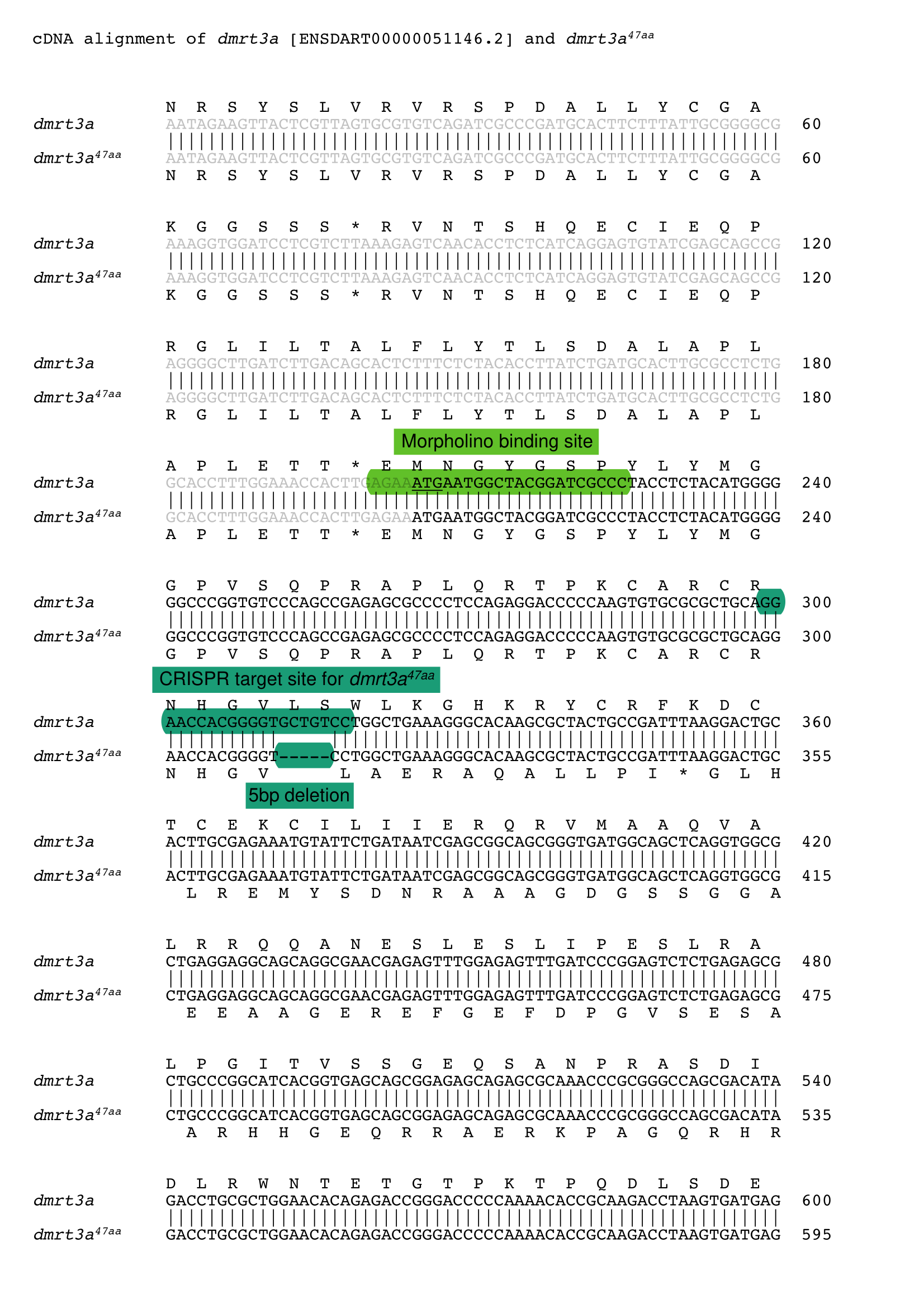

Supplement: Figure 1-1 — Alignment of dmrt3a cDNA from dmrt3a47aaand dmrt3aWT. Colors highlight the relevant sites for the generation of dmrt3a47aa, dmrt3aMO, and dmrt3a376aa. Download Figure 1-1, TIF file. [file enu-eN-CFN-0047-20-s02.tif]
