# Supplementary material for: Behavioral Characterization of dmrt3a Mutant Zebrafish Reveals Crucial Aspects of Vertebrate Locomotion through Phenotypes Related to Acceleration
Source: eNeuro. 2020 May 18;7(3):ENEURO.0047-20.2020. doi: 10.1523/ENEURO.0047-20.2020 (PMC7235372; doi:10.1523/ENEURO.0047-20.2020)
Supplement: Figure 2-1 — Number of replicates, statistical values, and p values for all the variables, developmental stages, and zebrafish models assessed during free swimming and escape trials. Download Figure 2-1, DOCX file. [file enu-eN-CFN-0047-20-s03.docx]

**Figure 2-1.** *Number of replicates, statistical values and p values for all the variables, developmental stages and zebrafish models assessed during free-swimming and escape trials.*

**3 dpf**

**4 dpf 5 dpf 6 dpf**

**10 dpf**

**22 dpf**

**Trial N(n) Stat p value Stat p value Stat p value Stat p value N(n) Stat p value N(n) Stat p value**

| ***dmrt3a 376aa*** |  | | | | | | | | | | | | | | | |
| --- | --- | --- | --- | --- | --- | --- | --- | --- | --- | --- | --- | --- | --- | --- | --- | --- |
| Total distance | Free-swimming | 2(182) | 2107.5 | 0.016 | 2936.0 | 0.611 | 4043.0 | 0.784 | 3500.0 | 0.910 | 2(128) | 0.511† | 0.610 |  |  |  |
| Nr. Movements | Free-swimming | 2(182) | 2129.0 | 0.200 | 2974.0 | 0.707 | 3998.0 | 0.688 | 3457.5 | 0.070 | 2(128) | 1981.0 | 0.866 |  |  |  |
| Mean movement duration | Free-swimming | 2(182) | 1735.5 | 0.270 | 2668.5 | 0.185 | 3850.0 | 0.414 | 3922.0 | 0.714 | 2(128) | 0.441† | 0.660 |  |  |  |
| Mean acceleration time | Free-swimming | 2(182) | 1650.0 | 0.127 | 2770.5 | 0.334 | 3843.0 | 0.403 | 3899.0 | 0.666 | 2(128) | 0.489† | 0.626 |  |  |  |
| Velocity while moving | Free-swimming | 2(182) | 1507.0 | 0.026 | 2455.0 | 0.037 | 4070.0 | 0.843 | 4019.0 | 0.929 | 2(128) | 0.700† | 0.486 |  |  |  |
| Max Acceleration | Free-swimming | 2(182) | 1814.5 | 0.000 | 3895.0 | 0.058 | 3895.0 | 0.490 | 4073.0 | 0.950 | 2(128) | 1999.0 | 0.935 |  |  |  |
| Max. Velocity | Free-swimming | 2(182) | 1773.0 | 0.000 | 2407.0 | 0.018 | 3921.0 | 0.537 | 3930.0 | 0.640 | 2(128) | 0.752† | 0.453 |  |  |  |
| Max. Acceleration | Escape | 2(182) | 2584.0 | 0.039 | 4312.5 | 0.443 | 4214.0 | 0.671 | 4107.0 | 0.639 | 2(128) | 1796.0 | 0.231 |  |  |  |
| Max. Velocity | Escape | 2(182) | 2721.0 | 0.111 | 4347.5 | 0.499 | 4220.0 | 0.528 | 4048.0 | 0.528 | 2(128) | 1824.0 | 0.287 |  |  |  |
| Time Accelerating | Escape | 2(182) | 0.535† | 0.593 | -0.693† | 0.489 | -0.465† | 0.643 | 0.879† | 0.380 | 2(128) | -0.692† | 0.490 |  |  |  |
| Total displacement | Escape | 2(182) | 2829.0 | 0.222 | 4477.5 | 0.735 | 4043.0 | 0.375 | 4273.0 | 0.989 | 2(128) | 1.423† | 0.157 |  |  |  |
| Cumulative Velocity | Escape | 2(182) | 2829.0 | 0.222 | 4477.5 | 0.735 | 4043.0 | 0.375 | 4273.0 | 0.989 | 2(128) | 1.423† | 0.157 |  |  |  |
| ***dmrt3a MO*** |  |  |  |  |  |  |  |  |  |  |  |  |  |  |  |  |
| Total distance | Free-swimming | 3(239) | 5322.0 | 0.119 | 5889.0 | 0.044 | 3983.0 | 0.000 | 4676.0 | 0.000 | 2(199) | 4031.0 | 0.025 | 1(30) | 0.128† | 0.899 |
| Nr. Movements | Free-swimming | 3(239) | 5385.5 | 0.154 | 5872.5 | 0.041 | 4103.0 | 0.000 | 4698.5 | 0.000 | 2(199) | 4198.0 | 0.066 | 1(30) | 0.019† | 0.985 |
| Mean movement duration | Free-swimming | 3(239) | 4977.0 | 0.100 | 6940.5 | 0.995 | 4164.0 | 0.000 | 5809.0 | 0.015 | 2(199) | 4087.0 | 0.046 | 1(30) | 109.0 | 0.885 |
| Mean acceleration time | Free-swimming | 3(239) | 4935.0 | 0.105 | 6825.0 | 0.903 | 4313.0 | 0.000 | 6137.0 | 0.068 | 2(199) | 4089.0 | 0.046 | 1(30) | 0.395† | 0.696 |
| Velocity while moving | Free-swimming | 3(239) | 5564.0 | 0.726 | 6836.0 | 0.837 | 4838.0 | 0.000 | 6965.0 | 0.783 | 2(199) | 4374.0 | 0.198 | 1(30) | 0.039† | 0.969 |
| Max. Acceleration | Free-swimming | 3(239) | 5000.0 | 0.025 | 6865.0 | 0.965 | 5018.0 | 0.000 | 6771.0 | 0.523 | 2(199) | 4939.0 | 0.990 | 1(30) | -0.500† | 0.621 |
| Max. Velocity | Free-swimming | 3(239) | 5325.0 | 0.121 | 6541.0 | 0.442 | 5449.0 | 0.005 | 6506.0 | 0.255 | 2(199) | 4884.0 | 0.883 | 1(30) | 0.456† | 0.652 |
| Max. Acceleration | Escape | 3(239) | 5863.5 | 0.189 | 4008.0 | 0.000 | 2861.0 | 0.000 | 2406.0 | 0.000 | 2(199) | 1.982† | 0.049 | 1(30) | 0.396† | 0.695 |
| Max. Velocity | Escape | 3(239) | 5804.0 | 0.152 | 5282.0 | 0.001 | 4044.0 | 0.000 | 3741.0 | 0.000 | 2(199) | 1.759† | 0.080 | 1(30) | 93.0 | 0.419 |
| Time Accelerating | Escape | 3(239) | 5313.5 | 0.015 | 4980.0 | 0.000 | 3969.0 | 0.000 | 4789.0 | 0.000 | 2(199) | 4881.5 | 0.919 | 1(30) | 94.0 | 0.439 |
| Total displacement | Escape | 3(239) | 5430.0 | 0.028 | 6985.0 | 0.812 | 5169.0 | 0.000 | 3.774† | 0.000 | 2(199) | 0.117† | 0.907 | 1(30) | 1.284† | 0.210 |
| Cumulative Velocity | Escape | 3(239) | 5430.0 | 0.028 | 6985.0 | 0.812 | 5169.0 | 0.000 | 3.774† | 0.000 | 2(199) | 0.117† | 0.907 | 1(30) | 1.284† | 0.210 |
| ***dmrt3a 47aa*** |  |  |  |  |  |  |  |  |  |  |  |  |  |  |  |  |
| Total distance | Free-swimming | 3(137) | 2167.0 | 0.713 | 1839.5 | 0.038 | 1851.0 | 0.043 | 2134.0 | 0.426 | 1(72) | 361.0 | 0.001 | 1(34) | 0.198† | 0.845 |
| Nr. Movements | Free-swimming | 3(137) | 2176.0 | 0.743 | 1779.0 | 0.020 | 1934.5 | 0.097 | -0.096† | 0.923 | 1(72) | 420.0 | 0.010 | 1(34) | -9.140† | 0.368 |
| Mean movement duration | Free-swimming | 3(137) | 1858.0 | 0.427 | 2114.0 | 0.547 | 2.530† | 0.013 | 1271.0 | 0.000 | 1(72) | 315.0 | 0.000 | 1(34) | 133.0 | 0.704 |
| Mean acceleration time | Free-swimming | 3(137) | 1728.5 | 0.154 | 2155.0 | 0.673 | 1851.0 | 0.042 | 1671.0 | 0.005 | 1(72) | 4.050† | 0.000 | 1(34) | -0.150† | 0.988 |
| Velocity while moving | Free-swimming | 3(137) | 1952.0 | 0.732 | 2192.0 | 0.797 | 2097.0 | 0.339 | 3.788† | 0.000 | 1(72) | 371.0 | 0.002 | 1(34) | 0.765† | 0.450 |
| Max. Acceleration | Free-swimming | 3(137) | 1970.0 | 0.215 | 2020.5 | 0.198 | 1.333† | 0.185 | 1851.0 | 0.043 | 1(72) | 2.492† | 0.015 | 1(34) | 0.498† | 0.622 |
| Max. Velocity | Free-swimming | 3(137) | 2031.0 | 0.332 | 2054.5 | 0.254 | 1985.0 | 0.149 | 1934.0 | 0.096 | 1(72) | 1.570† | 0.121 | 1(34) | 1.596† | 0.120 |
| Max. Acceleration | Escape | 3(137) | 2188.0 | 0.555 | 1379.0 | 0.001 | 728.0 | 0.000 | 559.5 | 0.000 | 1(72) | 7.464† | 0.000 | 1(36) | 102.0 | 0.058 |
| Max. Velocity | Escape | 3(137) | 2206.0 | 0.611 | 1526.0 | 0.006 | 911.0 | 0.000 | 947.5 | 0.000 | 1(72) | 7.478† | 0.000 | 1(36) | -8.310† | 0.412 |
| Time Accelerating | Escape | 3(137) | 2234.5 | 0.704 | 1587.0 | 0.014 | 1684.5 | 0.006 | 1216.0 | 0.000 | 1(72) | -3081† | 0.003 | 1(36) | -2.344† | 0.025 |
| Total displacement | Escape | 3(137) | 2214.0 | 0.637 | 1619.0 | 0.021 | 1084.0 | 0.000 | 1192.5 | 0.000 | 1(72) | 4.732† | 0.000 | 1(36) | 0.995† | 0.328 |
| Cumulative Velocity | Escape | 3(137) | 2214.0 | 0.637 | 1619.0 | 0.021 | 1084.0 | 0.000 | 1192.5 | 0.000 | 1(72) | 4.732† | 0.000 | 1(36) | 0.995† | 0.328 |

Stat generally indicates Mann-Whitney U. If dagger(†), Stat indicates Student t-test. Red values show the significant differences, p < 0.05

N(n) shows the number of performed trials (number of total animals used)
