# Supplementary material for: Behavioral Characterization of dmrt3a Mutant Zebrafish Reveals Crucial Aspects of Vertebrate Locomotion through Phenotypes Related to Acceleration
Source: eNeuro. 2020 May 18;7(3):ENEURO.0047-20.2020. doi: 10.1523/ENEURO.0047-20.2020 (PMC7235372; doi:10.1523/ENEURO.0047-20.2020)
Supplement: Figure 3-1 — Number of replicates, statistical values, and p values for all the variables assessed during coiling, tail kinematics, and maximum swim speed. Download Figure 3-1, DOCX file. [file enu-eN-CFN-0047-20-s04.docx]

**Figure 3-1.** *Number of replicates, statistical values and p values for all the variables assessed during coiling, tail kinematics and juveniles maximum speed.*

**Trial N(n) Stat p value**

| ***dmrt3a 47aa*** |  | | | |
| --- | --- | --- | --- | --- |
| Coiling duration | Coiling | 1(20) | -0.610† | 0.549 |
| Freq. Movements | Coiling | 1(20) | 0.378† | 0.710 |
| Mean change Intensity | Coiling | 1(20) | -0.605† | 0.553 |
| Nr. Half-beats | Tail kinematics - Slow | 2(200) | 3839.5 | 0.009 |
| Cumulative duration | Tail kinematics - Slow | 2(200) | 4024.0 | 0.033 |
| Cumulative tail trajectory | Tail kinematics - Slow | 2(200) | 3856.0 | 0.011 |
| Mean tail trajectory | Tail kinematics - Slow | 2(200) | 4007.0 | 0.030 |
| Mean tail velocity | Tail kinematics - Slow | 2(200) | 4005.5 | 0.029 |
| Max. Deflection | Tail kinematics - Slow | 2(200) | 4864.0 | 0.954 |
| Max. Curvature beats | Tail kinematics - Slow | 2(200) | 4642.0 | 0.544 |
| Variance of half-beats period | Tail kinematics - Slow | 2(200) | 4423.0 | 0.251 |
| Nr. Half-beats | Tail kinematics - Fast | 2(81) | 648.0 | 0.558 |
| Cumulative duration | Tail kinematics - Fast | 2(81) | 681.0 | 0.845 |
| Cumulative tail trajectory | Tail kinematics - Fast | 2(81) | 608.0 | 0.347 |
| Mean tail trajectory | Tail kinematics - Fast | 2(81) | 549.0 | 0.123 |
| Mean tail velocity | Tail kinematics - Fast | 2(81) | 420.0 | 0.004 |
| Max. Deflection | Tail kinematics - Fast | 2(81) | 694.0 | 0.951 |
| Max. Curvature beats | Tail kinematics - Fast | 2(81) | 688.0 | 0.902 |
| Variance of half-beats period | Tail kinematics - Fast | 2(81) | 633.5 | 0.452 |
| Max. speed | Juveniles Max. Speed | 3(123) | 1470 | 0.030 |

Stat generally indicates Mann-Whitney U test. If dagger(†), Stat indicates Student t-test. Red values show the significant differences, p < 0.05

N(n) shows the number of performed trials (number of total animals used) for coiling

analyses or performed trials (number of analysed movements) for tail kinematics.
